# Li-Fraumeni syndrome: not a straightforward diagnosis anymore—the interpretation of pathogenic variants of low allele frequency and the differences between germline PVs, mosaicism, and clonal hematopoiesis

**DOI:** 10.1186/s13058-019-1193-1

**Published:** 2019-09-18

**Authors:** Felipe Batalini, Ellie G. Peacock, Lindsey Stobie, Alison Robertson, Judy Garber, Jeffrey N. Weitzel, Nadine M. Tung

**Affiliations:** 1 Division of Hematology and Oncology, Beth Israel Deaconess Medical Center, Harvard Medical School, 330 Brookline Ave, Boston, MA 02215 USA; 2 Dana-Farber Cancer Institute, 450 Brookline Ave, Boston, MA 02215 USA; 3 Center for Cancer Genetics and Prevention, Harvard Medical School, Dana-Farber Cancer Institute, 450 Brookline Ave, Boston, MA 02215 USA; 4 Division of Clinical Cancer Genomics, City of Hope Cancer Center, 1500 East Duarte Road, Duarte, CA 91010 USA; 5 Cancer Risk and Prevention Program, Beth Israel Deaconess Medical Center, Harvard Medical School, 330 Brookline Ave., Boston, MA 02215 USA

**Keywords:** Mutation, Pathogenic variant, TP53, Li-Fraumeni syndrome, Hereditary breast cancer, Clonal hematopoiesis, Mosaicism, Low allele frequency

## Abstract

The introduction of next-generation sequencing has resulted in testing multiple genes simultaneously to identify inherited pathogenic variants (PVs) in cancer susceptibility genes. PVs with low minor allele frequencies (MAFs) (< 25–35%) are highlighted on germline genetic test reports. In this review, we focus on the challenges of interpreting PVs with low MAF in breast cancer patients undergoing germline testing and the implications for management.

The clinical implications of a germline PV are substantial. For PV carriers in high-penetrance genes like *BRCA1*, *BRCA2*, and *TP53*, prophylactic mastectomy is often recommended and radiation therapy avoided when possible for those with Li-Fraumeni syndrome (LFS). For germline PV carriers in more moderate-risk genes such as PALB2, *ATM*, and *CHEK2*, annual breast MRI is recommended and prophylactic mastectomies considered for those with significant family histories. Detection of PVs in cancer susceptibility genes can also lead to recommendations for other prophylactic surgeries (e.g., salpingo-oophorectomy) and increased surveillance for other cancers. Therefore, recognizing when a PV is somatic rather than germline and distinguishing somatic mosaicism from clonal hematopoiesis (CH) is essential. Mutational events that occur at a post-zygotic stage are somatic and will only be present in tissues derived from the mutated cell, characterizing classic mosaicism. Clonal hematopoiesis is a form of mosaicism restricted to the hematopoietic compartment.

Among the genes in multi-gene panels used for germline testing of breast cancer patients, the detection of a PV with low MAF occurs most often in *TP53*, though has been reported in other breast cancer susceptibility genes. Distinguishing a germline *TP53* PV (LFS) from a somatic PV (*TP53* mosaicism or CH) has enormous implications for breast cancer patients and their relatives.

We review how to evaluate a PV with low MAF. The identification of the PV in another tissue confirms mosaicism. Older age, exposure to chemotherapy, radiation, and tobacco are known risk factors for CH, as is the absence of a LFS-related cancer in the setting of a *TP53* PV with low MAF. The ability to recognize and understand the implications of somatic PVs, including somatic mosaicism and CH, enables optimal personalized care of breast cancer patients.

## Introduction

Current estimates are that 5–10% of breast cancer results from an inherited gene pathogenic variant (PV) (Table 1). As such, taking a detailed family history is a key component of the initial visit with the oncology practitioner. The recognition of hereditary patterns or key features of inherited predisposition such as early age at onset or multiple primary tumors triggers the evaluation for familial cancer syndromes. The most common of these is the hereditary breast and ovarian cancer syndrome (HBOC), most often associated with pathogenic variants (PV) in *BRCA1* or *BRCA2* [1–5]. PVs in other high-risk cancer susceptibility genes like *TP53* (Li-Fraumeni Syndrome; LFS), while not as common, have important clinical significance. The diagnosis of LFS impacts management because of the high risk of bilateral breast cancer and potential for in-field new primary tumors after radiation therapy, and results in recommendations for extensive cancer surveillance for breast and other cancers in the patient and at-risk relatives. Genetic testing is the final diagnostic step to confirm inherited risk and, if a PV is identified, can affect management of the patient and enable cascade genetic testing of relatives.

**Table 1:** Glossary

1. Variant: any change in DNA sequence.	
2. Pathogenic or likely pathogenic variant (PV): change in DNA sequence that alters the function of a gene (i.e., a variant that predisposes to disease, in this case cancer). Often referred to as a “mutation.”	
3. Germline genetic testing: testing germline DNA (generally blood leukocytes) for inherited PVs.	
4. Somatic genomic testing of tumor: testing a tumor for variants and PVs, either by tumor biopsy or circulating free DNA.	
5. Minor allele frequency (MAF): the relative frequency of the allele with the variant (or PV) in a population, expressed as a fraction or percentage. A low MAF (< 25–35%, depending on the commercial laboratory) is suggestive of a somatic, rather than germline, PV.	
6. Germline PV: a heritable change in the DNA that is present in a germ cell (egg or in the sperm). When transmitted to a child, a germline PV is incorporated in every cell of the offspring.	
7. Somatic PV: a post-zygotic alteration in DNA that occurs during embryonic development or later in a person’s life. Somatic PVs can occur in any of the cells of the body, and unless they also involve the germ cells (sperm or egg), they are not heritable. Tumor-specific PVs are a type of somatic PV.	
8. Mosaicism: the presence of at least two cell lines differing in genotype, derived from the same zygote.	
9. Somatic (classic) mosaicism: the somatic cells of the body are of more than one genotype. This results from a PV that occurred post-zygote or a nondisjunction event in an early mitosis.	
10. Clonal hematopoiesis (CH): a PV in a subpopulation (clone) of the hematopoietic cells but not in other tissues.	
11. Germline (gonadal) mosaicism: a type of genetic mosaicism where more than one set of genetic information is found in an individual, specifically within their gamete cells due to a PV that occurred after conception. The offspring will have a germline PV with a variant allele frequency of ~ 50%. The parent with the gamete PV will not appear to have a PV from standard genetic testing. The offspring will appear to have a de novo germline PV.	
12. De novo PV: a PV that is present for the first time in an individual but is not detectable in the blood of either parent. This can be the result of a PV in a gamete cell (egg or sperm) of one parent (gonadal mosaicism), or that arises in the fertilized egg itself during very early embryogenesis.	
13. Depth of sequencing: coverage (or depth) in DNA sequencing is the number of unique reads that include a given nucleotide, a particular DNA sequence. Deep sequencing refers to the general concept of aiming for a high number of unique reads of each region of a sequence.	
14. Proband: a person who serves as the starting point for genetic evaluation of a family.	

The introduction of next-generation sequencing (NGS) coupled with the continuous decrease in the cost of genetic testing has resulted in the common practice of testing multiple genes simultaneously to identify inherited PVs in cancer susceptibility genes. NGS is not only more efficient but also more sensitive in detecting genetic variants due to higher depth of coverage [6]. The use of multi-gene panels by breast oncologists to detect germline PVs has become routine, and almost every cancer panel includes *TP53* [7]. In addition, commercially available direct-to-consumer genetic test products are now available, allowing individuals to sequence their own genome and bring the results to physicians for interpretation.

It is therefore increasingly important that oncology practitioners are comfortable interpreting genetic test results for inherited PVs. NGS technology has allowed quantifying minor allele frequencies (MAFs), which has become a key factor for the diagnostic laboratory in interpreting genetic test results. MAF refers to the fraction of alleles at the specific locus that carry the specific variant. PVs are assumed to be of germline origin when the allele frequency is approximately 50%, but a range between 30 and 70% is commonly accepted as representing a heterozygous PV [8]. The implications of detecting a germline PV are extremely significant and often lead to the recommendation of prevention strategies that can include risk-reducing surgeries, as well as testing of relatives who may also carry the PV.

While the MAF is not routinely reported with commercial hereditary cancer panels, low variant allele frequencies (< 25–35% depending on the testing company) are often highlighted as a comment on a genetic test report. However, the etiology and clinical implications of PVs with low allele frequency are less clear. For breast cancer patients who undergo germline genetic testing, a low MAF is increasingly encountered. Weitzel et al. reported that MAFs < 25% were responsible for 20% of “positive” *TP53* pathogenic variants [7]. While the detection of a PV with a low MAF occurs with higher frequency in several hematologic malignancy-related genes [9], with the current multi-gene panels used for breast cancer patients, a low MAF is most often detected in *TP53*, but has also been described in other breast cancer susceptibility genes [10, 11].

The finding of a PV with a low MAF on a hereditary cancer panel can be due to somatic PVs, namely somatic mosaicism, clonal hematopoiesis (CH), technical interference in the assay, or tumor PVs detected in the blood from a circulating hematologic malignancy or from solid tumors. In fact, Coffee et al. reported that 38.8% of *TP53* pathogenic variants detected using one commercially available hereditary cancer panel were, in fact, likely somatic and not germline variants [10].

In this review, we focus on the challenges of interpreting pathogenic variants with low MAF in patients with breast cancer who undergo germline genetic testing and the implications for management.

### The role of germline testing

The use of multi-gene panels for germline testing has become routine in the management of breast cancer. Approximately 10% of women with newly diagnosed breast cancer have a PV in a high- or moderate-penetrance cancer susceptibility gene, the majority of which are in breast cancer susceptibility genes [12, 13].

The clinical implications of a germline PV are significant. For PV carriers in high-penetrance genes—*BRCA1*, *BRCA2*, *CDH1*, PTEN, and *TP53*—prophylactic contralateral mastectomy is often recommended. For PV carriers in more moderate-risk genes such as PALB2, *ATM*, and *CHEK2*, increased surveillance with annual breast MRI is recommended and prophylactic mastectomies are often considered in those with significant family histories. Detection of PVs in other cancer susceptibility genes can lead to recommendations of prophylactic surgeries, such as salpingo-oophorectomy, or increased surveillance for other cancers [14].

The selection of systemic therapy for breast cancer may also be impacted by the detection of a germline BRCA PV. For example, PARP inhibitors (olaparib and talazoparib) increase progression-free survival compared with chemotherapy for germline BRCA carriers with metastatic disease and are being investigated in the early setting [15, 16]. Breast cancer patients with LFS are usually treated with mastectomy to avoid potential radiation-induced malignancies and can be enrolled in clinical trials of aggressive screening, given the high lifetime risk of cancers in multiple organs. Importantly, identifying germline PVs in a breast cancer patient triggers cascade genetic testing of relatives. Identifying PVs in unaffected relatives affords the opportunity for early detection through increased surveillance and prevention.

Interestingly, recent studies have reported that despite current guidelines (e.g., NCCN), a large proportion of patients who harbor germline PVs are not being identified [17, 18]. As a result, some guidelines now recommend universal genetic testing for all breast cancer patients. As the threshold for germline testing decreases, a larger number of breast cancer patients will be tested, and an increasing number of PVs with a low MAF will be identified.

### Classic (somatic) mosaicism (Fig. 1)

The zygote is the earliest developmental stage of a human cell, which is formed by the fertilization event between parental gametes, and its genome is thus a combination of the parental DNA. When a PV occurs in the parent’s gamete cells (gonadal mosaicism) or when the PV arises in the unicellular zygote, all daughter cells carry that PV. This defines a new (de novo) PV that exists in the individual but is not identified in either parent. These are heritable for the individual’s offspring (50% probability), and there is a small chance (usually counseled as ~ 5%) that a sibling also inherited the PV, depending on what fraction of the parental gonadal cells has the PV.

**Fig. 1:**
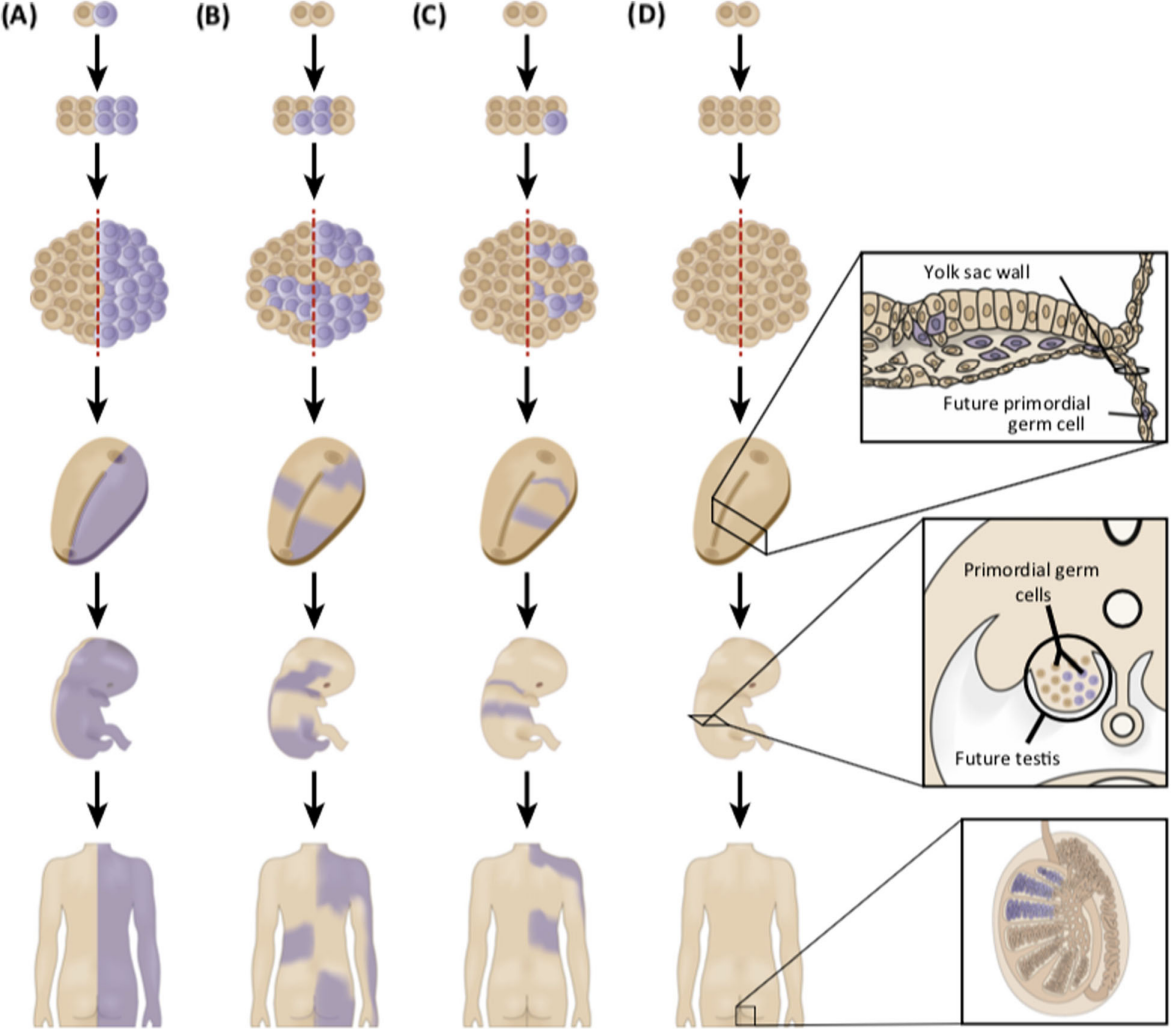
Classic (somatic) mosaicism: The timing of post-zygotic mutation influences the distribution of mutant cells. **a** Mutations that occur during the first mitosis result in approximately half of the individual being affected. **b** Mutations that occur before left–right determination can affect both sides of the individual, including one or both gonads. **c** Mutations that arise after the determination of the two sides of the embryo can be confined to only one side of the individual. Only one gonad is likely to be affected. **d** Mutations that occur after differentiation of primordial germ cells will be absent from somatic tissues (reprinted with permission of the author [19, 20])

In contrast, if the mutational event happens at a post-zygotic stage, the PV will only be present in tissues derived from the mutated cell, characterizing classic mosaicism. Attention should be paid to the term *mosaicism* in the literature; while it is often used to refer to classic post-zygotic mosaicism, which develops from a PV very early in embryogenesis, it can also refer to somatic PVs acquired later, such as clonal hematopoiesis, i.e., somatic PVs that develop in hematopoietic cells only leading to clonal expansion in the blood, or even in the post allogeneic bone marrow transplant setting (also referred to as chimerism). In a classic post-zygotic mosaic, the frequency of mutant reads would be similar across different tissues. Both classic mosaicism and clonal hematopoiesis typically result in a PV with a low MAF.

The evaluation of suspected classic mosaicism varies widely across institutions. Genotyping of fibroblast (skin punch biopsy) or more recently hair follicle (eyebrow) samples are commonly used options [7]. In a patient found to have a *TP53* PV with a low MAF by germline testing, the presence of the PV in any of these tissues, would confirm the diagnosis of classic mosaicism.

The clinical implications of classic mosaicism are unclear since it is uncertain which (embryonic) tissue layers harbor the PV; such knowledge would ideally inform the appropriate surveillance strategy. Currently, there is no defined protocol to assess the distribution of tissues that harbor the PV. Therefore, it is unclear whether patients with *TP53* classic mosaicism (i.e., patients with likely post-zygotic mosaic LFS) should be managed the same as patients with true LFS. Currently, the same surveillance strategies are often recommended though data to support this practice are lacking. For a breast cancer patient found to have classic *TP53* mosaicism, the presumption is that the breast tissue carries the PV, although analysis of normal breast tissue would be required to confirm this. Somatic testing of the tumor would be expected to identify the PV with a high MAF since loss of heterozygosity (LOH) for the wild type *TP53* allele would be anticipated in the development of the cancer, although data to support this assumption is scant. In addition, some PVs occur in domains that result in so-called dominant negative effects, and may not be accompanied by LOH since LOH is not required for the PV to have a deleterious effect.

A potential strategy to obtain a more comprehensive profiling of mutated tissues in a *TP53* mosaic would be to employ tissue-specific analysis. Early in embryonic development, gastrulation results in the formation of the three germ layers: endoderm, mesoderm, and ectoderm. The epiblast gives origin to the ectoderm and endoderm. The mesoderm then develops from the interaction of the ectoderm and endoderm. Germline testing is usually performed using leukocytes (mesodermal origin) extracted from the buffy coat of peripheral blood after centrifugation. In the case of a low MAF PV, confirmatory testing to identify that PV in tissues other than hematopoietic cells can be performed using fibroblasts (mesodermal origin) from skin punch biopsy. Cells obtained from buccal swabs or eyebrow (all ectodermal origin) samples can also be used. Through special techniques to separate the different skin layers (e.g., use of suction blisters and salt splitting performed on fresh tissue sent to pathology), skin biopsy could assess both mesoderm and ectoderm since the epidermis is derived from the ectoderm, and both dermis and lower layers of the skin are derived from the mesoderm. Endodermal tissues could be sampled from the colon, bronchus, or urinary bladder. Ideally, obtaining tissues from all three germ layers would provide a more complete understanding of the distribution of PVs. However, mosaicism can also develop later in embryonic development and lead to PVs that are limited to tissue that cannot be sampled. If a *TP53* PV were identified in an ectoderm-derived tissue such as the epidermis or hair and in a mesoderm-derived tissue such as blood, it would be reasonable to infer that it is also present in the endoderm.

Whereas a germline PV carrier has a 50% chance of passing the PV to an offspring, in a classic mosaic, there is no way to know whether the PV is present in the germ cells of that individual and in what ratio. Therefore, the risk for offspring to inherit the PV is unknown, but could be as high as 50%. Whether the PV is present in the germ cells of a classic mosaic would depend on when in embryogenesis the PV occurred—before or after the creation of the primordial germ cells.

### Clonal hematopoiesis (CH)

During hematopoiesis, replicative division and regeneration by the hematopoietic stem/progenitor cell (HSPC) over decades leads to the accumulation of rare random PVs that trigger the activation of cell machinery responsible for proliferation and self-renewal. This confers a survival advantage over other HSPC, resulting in clonal expansion of mutated cells, without necessarily resulting in the development of overt hematologic malignancy.

Clonal expansion in the circulating hematopoietic cells has been studied and reported under multiple terms, including clonal hematopoiesis of indeterminate potential (CHIP), aberrant clonal expansion (ACE), and age-related clonal hematopoiesis (ARCH). CHIP is defined as the presence of the clonal expansion without clinically significant cytopenias or dysplastic hematopoiesis [9]. The prevalence of clonal hematopoiesis varies according to the way pathogenic PVs are defined, which variant allele fractions are included and depth of assay coverage.

Large cohort studies have demonstrated that CH affects a significant portion of the healthy population and its prevalence increases with age [21–24]. Jaiswal et al. analyzed whole exome sequencing (WES) in 17,182 individuals derived from 22 population-based cohorts and sought somatic PVs in 160 genes that are recurrently mutated in hematologic malignancies. They found that CH is rare in persons younger than age 40 but that the frequency increased with age. The frequency of CH was 9.5%, 11.7%, and 18.4% in individuals aged 70–79, 80–89, and 90–108, respectively. The majority of PVs occurred in three genes, DNMT3A, TET2, and ASXL1. They also showed that these PVs are not transient by including a small number of patients with serial blood collections over 4 to 8 years. In this study, MAF as low as 3.5% was included and the sequencing depth was 84 reads. Likewise, Genovese et al. found similar results in 12,380 persons unselected for cancer or hematologic phenotype. They observed CH in 10% of persons older than 65 years of age but in only 1% younger than age 50 [24].

CH has been reported among breast cancer susceptibility genes, though has most frequently been reported in genes known to be associated with leukemia. This may be due to differences in genes included in panels used to test hematologic cancer and breast cancer patients. Steensma et al. noted that 80% of the PVs causing CH involve 19 genes [9]. From these, only *TP53* is commonly included in the panels used to test patients with breast cancer [25]. While *TP53* is the breast cancer risk gene most reported to develop CH, less commonly, clonal hematopoiesis can occur in other breast cancer susceptibility genes such as *ATM*, *CHEK2* [10], PTEN, NF-1, and even *BRCA1* and *BRCA2* [11, 26]. Coffee et al. reported PVs with a MAF 10–30% in *CHEK2* (*n* = 27) and *ATM* (*n* = 20) in more than 220,000 individuals undergoing germline genetic testing [10]. Ptashkin et al. sequenced tumor and matched peripheral blood in 17,469 patients with solid tumors and reported a case in which a patient had a *BRCA2* PV with a MAF of 34% from a peripheral blood sample with a lower MAF of 11% in the tumor. Analysis of the saliva, buccal swab, and colon confirmed that the variant was restricted to the hematologic compartment, likely indicating CH. These authors also found CH in a single case each involving *BRCA1* and PTEN and in three cases involving NF-1 [11].

Ruark et al. demonstrated a potential connection between somatic mosaic *PPM1D* PVs and a predisposition for breast and ovarian cancer, identifying *PPM1D* PVs in 25 of 7781 cases of breast or ovarian cancer but in only one of 5861 controls, *p* = 1.12 × 10^−5^. The PVs were mosaic in lymphocyte DNA with the mutant allele consistently and considerably lower than the *PPM1D* wild type allele. Family studies were also consistent with mosaicism since none of the relatives analyzed carried the PV. In addition, the authors demonstrated that two separate PV carriers each had two offspring who had inherited different maternal *PPM1D* alleles, but that neither carried the maternal PV, and concluded that the PVs were either not present or mosaic in the gamete of the women with the PVs [27]. Of note, the potential contribution of prior cancer treatment to the development of somatic *PPM1D* somatic PVs was not considered in this study.

In addition to age, chemotherapy has also been associated with CH. Swisher et al. evaluated the impact of chemotherapy on the development of CH using the BROCA panel (65 genes) in 686 women with ovarian carcinoma and showed that chemotherapy is associated with the development of somatic mosaic PVs in the *PPM1D* gene. *PPM1D* PVs were significantly associated with chemotherapy (*p* < 0.001) and with the number of prior regimens. The odds ratio for a PV was 4.82 (95% CI, 1.43–16.18) in chemotherapy-treated women and 17.24 (95% CI, 6.8–43.69) in those with recurrent platinum-resistant disease. Among women treated with chemotherapy, *PPM1D* PVs were significantly more frequent with age. Somatic PVs were not confirmed in any other gene on the BROCA panel except *TP53*. However, in this study, CH in *TP53* was not associated with chemotherapy exposure or increased age. Of note, of the 11 cases with *TP53* somatic PVs, five also had a PV in *PPM1D*. In 17 patients with either a *TP53* or *PPM1D* somatic PV detected in blood, neither was detected in paired tumor specimens, confirming the diagnosis of CH. Serial testing of a few patients in the study demonstrated the emergence of somatic PVs in the *PPM1D* and *TP53* genes after the use of chemotherapy [28].

Coombs et al. also found an association of CH with chemotherapy, as well as radiation therapy and tobacco use. They found CH in 25% of 8810 patients with solid tumors using the MSK-IMPACT panel with 410 cancer-associated genes and including a MAF greater than 1% using paired tumor and peripheral blood [29]. PVs in the gene DNMT were most common, but PVs in *PPM1D*, *ATM*, and *TP53* were among the six genes most commonly mutated. CH was significantly associated with chemotherapy for *PPM1D* (*p* < 0.001) and to a lesser degree for *TP53* (*p* = 0.047), and with radiation therapy for both genes (*p* < 0.001). Of note, the median MAF for CH was 4.4%, lower than the reporting threshold for most commercial laboratories. CH was also significantly associated with age (*p* < 0.01) and prior tobacco use (*p* < 0.01) [21, 28, 29].

CH can indeed be a confounder of tumor genomic testing. Coombs et al. noted that 65% of tumors undergoing NGS had PVs in genes frequently altered in CH and CH accounted for 8% of PVs identified [30]. Likewise, Ptashkin et al. found that 5.2% of patients with solid tumors would have had at least one CH-associated PV erroneously classified as tumor-derived in the absence of matched blood sequencing. In the case of *TP53*, 4% of PVs found through tumor testing were due to CH [11]. Likewise, CH may confound interpretation of circulating free DNA (cfDNA) whether in patients with known cancer or in an attempt to screen for occult cancer [31]. Phallen et al. analyzed 58 cancer-related genes and found genomic changes related to CH in 16% of 44 healthy individuals [32]. Hu et al. identified PVs in JAK2, *TP53*, and even KRAS, which were due to CH and identified through analysis of cfDNA in patients with advanced cancer. Thus, clinicians ordering plasma genotyping must be aware of the possibility that PVs found may not represent a tumor genotype, especially in genes mutated in CH [33]. Paired leukocyte genotyping may be necessary so that CH-derived PVs are not misdiagnosed as malignancy or as representing the genotype of a known cancer. *Fortunately*, even though circulating tumor DNA (ctDNA) can potentially confound results from germline testing, ctDNA levels tend to be well below laboratory reporting levels [31].

CH has been associated with increased risk of hematologic malignancies, cardiac disease, and even solid tumors as well as increased mortality. Jaiswal et al. found that the presence of CH was associated with an increased risk of hematologic malignancies (hazard ratio (HR), 11.1; 95% confidence interval [CI], 3.9 to 32.6), cardiovascular disease (HR, 2.0; 95% CI, 1.2 to 3.4), and all-cause mortality (HR, 1.4; 95% CI, 1.1 to 1.8) [22]. Likewise, Genovese et al. found similar results for the risk of hematologic malignancies and all-cause mortality in 12,380 healthy individuals and suggested that the PVs play a direct role in future hematologic cancer development [24]. In patients with solid tumors, Coombs et al. observed that cancer patients harboring CH not only were at higher risk of developing hematologic malignancies, but also had worse survival outcomes [29]. Using data from 13 genome-wide association studies and more than 50,000 cases, Jacobs et al. found that mosaic chromosomal (> 2 Mb) abnormalities were more common in persons who later developed solid tumors [0.97% compared to 0.74% in cancer-free individuals; odds ratio (OR) = 1.25; *p* = 0.016] and had a stronger association with cases in which DNA was collected before diagnosis or treatment (OR = 1.45; *p* = 0.0005) [21].

Currently, there are no guidelines for managing patients with CH. It has been recommended that the CBC be evaluated and the presence of cytopenia warrants referral for specialized evaluation. In the setting of cytopenia, the analysis of combination of PVs and the MAF may indicate a high positive predictive value for development of an overt hematologic malignancy [34]. Bolton et al. suggested that the use of specific criteria for reporting CH to patients, which included abnormal indices in CBC, MAF ≥ 10%, or the presence of more than one PV, would indicate the need for further evaluation [35].

### The diagnosis of the Li-Fraumeni syndrome

#### *TP53* PV with MAF suggestive of germline origin (> 30%)

Classically, Li-Fraumeni syndrome (LFS) has been diagnosed with the use of the Chompret criteria to identify patients who warrant genetic testing for the presence of a germline *TP53* PV (Table 2). This has been revised in 2015 to include breast cancer diagnoses under age 31. Furthermore, most women with breast cancer and LFS have HER2-positive breast tumors [37]. The implications of finding a *TP53* PV include more aggressive cancer surveillance using breast MRI, as well as frequent colonoscopy initiated at an early age. Ongoing research trials are evaluating the benefit of whole-body MRI in patients with LFS given the increased risk of cancers in multiple organs and participation in clinical trials is strongly encouraged. For women with LFS who have breast cancer, mastectomy is favored over lumpectomy in an attempt to avoid ionizing radiation, which may increase the risk of secondary malignancies, including sarcoma [38–42].

**Table 2:** 2015 Chompret criteria for Li-Fraumeni syndrome and germline *TP53* mutation screening [36]

Must meet at least one criteria	
1. Familial presentation	
Proband with tumor belonging to Li-Fraumeni syndrome (LFS) tumor spectrum (e.g., soft tissue sarcoma, osteosarcoma, CNS tumor, breast cancer, adrenocortical carcinoma, leukemia, bronchoalveolar lung cancer) before age 46 years AND at least one first- or second-degree relative with an above LFS tumor (except breast cancer if proband has breast cancer) before age 56 years or with multiple tumors at any age	
2. Multiple primary tumors	
Proband with multiple tumors (except multiple breast tumors), two of which belong to LFS tumor spectrum and the first occurring before age 46 years	
3. Rare tumors	
Patient with adrenocortical carcinoma, choroid plexus carcinoma, or rhabdomyosarcoma of embryonal anaplastic subtype, irrespective of family history	
4. Early-onset breast cancer	
Breast cancer before age 31 years	

With the widespread use of large gene panels that include the *TP53* gene, patients without a family history of LFS and who do not meet the Chompret criteria are being identified with *TP53* PVs, often with a MAF that is compatible with germline inheritance (i.e., ~ 50%). Multi-gene panel testing has identified these unexpected *TP53* PV carriers, demonstrating a broader clinical phenotype of LFS [43]. Approximately 14% of patients with *TP53* PVs that appear to be germline by MAF are considered “de novo” since the PV is not identified in either parent [44]. “De novo” PVs can be due to gonadal mosaicism, resulting from a *TP53* PV that occurs in the gamete of one parent. The penetrance of the *TP53* PV in patients with a genotype of LFS without the representative personal or family history of cancer is unknown. In the absence of data from longitudinal studies, individuals with “de novo” germline PVs are currently managed the same as other individuals with LFS and a concordant family history.

Complicating the interpretation of “de novo” *TP53* PVs that appear to be germline, Coffee et al. have reported that somatic PVs with a MAF ~ 50% can also result from CH [10]. This was evidenced by serial testing of a patient with a *TP53* PV in whom the MAF increased from 30 to 45% over 3 months. Paired skin testing confirmed the absence of *TP53* PV in other tissues [10]. The frequency of CH with MAF > 30% is yet unknown.

#### *TP53* PVs with low MAF (< 30%)

**Fig. 2:**
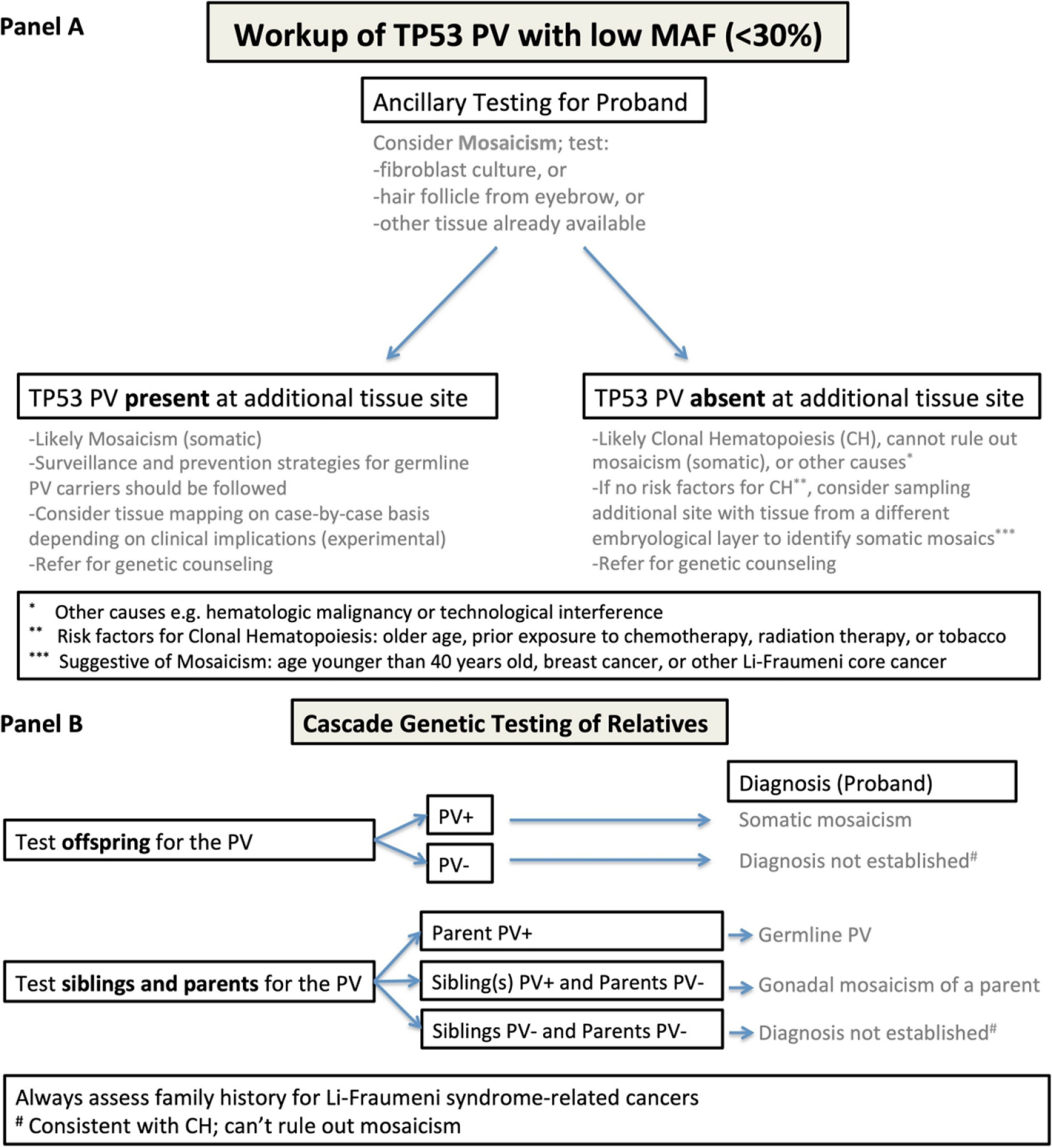
Recommended workup for a pathogenic or likely pathogenic variant (PV) with low minor allele frequency (MAF) in TP53. **a** Workup to distinguish somatic mosaicism from clonal hematopoiesis (CH). **b** Interpretation of genetic testing results in relatives

Just as challenging is the interpretation of a *TP53* PV with a low allele frequency (< 30%) (Fig. 2). The main differential is between classic (somatic) mosaicism and CH. Confirmation of somatic mosaicism can be achieved by identifying the *TP53* PV in a second tissue through fibroblast cultures, or from genotyping of cells obtained from buccal swabs, or eyebrow samples. Identification of the *TP53* PV through any of these means confirms mosaicism, while the failure to identify the PV does not exclude mosaicism. The identification of classic mosaicism must be followed by genetic counseling to discuss surveillance strategies for the affected individual and testing of offspring. Additionally, identifying the PV in another family member would exclude CH; therefore, it is important to test other family members including offspring, regardless of failing to identify the PV in a different tissue type, as the possibility of mosaicism remains.

Prior to 2013, when commercial multi-gene panel testing was initiated for germline testing of cancer susceptibility genes, no patients at our academic center had ever been identified with a PV with a MAF < 30% in a cancer susceptibility gene. Since 2016, 10 patients with a low MAF have been identified: 9 in *TP53*, 1 in *ATM*, and 1 in *NF1 (total of 11 PVs.)* Evaluation of the four individuals with *TP53* PVs using skin biopsy revealed that one had the PV identified in skin fibroblasts, confirming mosaicism, two had no PV, and one had a biopsy that failed fibroblast culture. Commercial genetic testing companies generally do not report a MAF < 10%.

## Conclusions

In comparison with Sanger sequencing, NGS with multi-gene panels has increased the sensitivity of germline genetic testing, leading to an increased ability to identify patients with somatic mutations. For the oncology practitioner ordering and interpreting germline genetic test reports, the interpretation of a PV with low MAF must consider the possibility of a somatic PV, including classic mosaicism and CH, as well as a germline PV.

Paired analysis from another tissue using cultured fibroblasts, buccal cells, or eyebrow hair can distinguish CH from classic mosaicism. The presence of the PV in a different tissue confirms classic mosaicism. CH is more common in older patients, with chemotherapy or radiation therapy, with tobacco use, and in patients with cancer independent of previous treatment. The practitioner should be suspicious that a *TP53* PV is due to CH if the cancer phenotype is significantly discordant (e.g., very late-onset cancer, especially if not a core LFS tumor). Among breast cancer susceptibility genes, CH has most frequently been reported in *TP53*, though has also occurred with *ATM*, *CHEK2*, PTEN, *NF*, and even *BRCA1* and *BRCA2*.

The detection of CH confounding germline and tumor somatic testing will be increasingly important for optimal selection of patients for clinical trials and for approved targeted therapies. Paired genotyping of tumor and leukocytes may be important to identify CH; the presence of a higher MAF in the blood than in tumor would strongly suggest this diagnosis [11]. Misinterpretation of genetic results may lead to inaccurate decision-making, such as the use of a therapy targeted to a PV that may not be present in the tumor.

The threshold for genetic testing is constantly decreasing and a greater proportion of breast cancer patients are undergoing germline testing. An accurate interpretation of genetic test results and the ability to recognize and understand the implications of somatic PVs including somatic mosaicism and CH is required for the appropriate personalized care of our patients.

## Data Availability

No datasets were generated or analyzed during the current study.
